# Neuroprotective effects of lixisenatide against propagation of α-synuclein pathology in Parkinson’s disease

**DOI:** 10.4103/NRR.NRR-D-24-00941

**Published:** 2025-03-25

**Authors:** Shangqi Sun, Liqin Huang, Gege Jiang, Guanfeng Xie, Xiaoyi Li, Xiufeng Wang, Hongxiu Guo, Cailin Wang, Siyi Zheng, Gang Li, Jing Xiong

**Affiliations:** 1 Department of Neurology, Union Hospital, Tongji Medical College, Huazhong University of Science and Technology, Wuhan, Hubei Province, China; 2 Department of Neurology, Renmin Hospital of Wuhan University, Wuhan, Hubei Province, China; 3 Taikang Center for Life and Medical Sciences, Wuhan University, Wuhan, Hubei Province, China

**Keywords:** α-synuclein propagation, disease-modifying treatment, glucagon-like peptide-1 receptor agonists, lixisenatide, lymphocyte-activation gene 3, mitochondrial dysfunction, Parkinson’s disease

## Abstract

Glucagon-like peptide-1 receptor agonists, originally developed for the treatment of type 2 diabetes mellitus, have been suggested as a potential disease-modifying treatment for Parkinson’s disease. Some clinical trials of glucagon-like peptide-1 receptor agonists have demonstrated that they can alleviate motor dysfunction and improve quality of life for patients with Parkinson’s disease. However, the mechanisms underlying the neuroprotective effects of glucagon-like peptide-1 receptor agonists have yet to be elucidated. In this study, we used α-synuclein preformed fibrils to generate *in vitro* and *in vivo* models of Parkinson’s disease and investigated the effects of a short-acting glucagon-like peptide-1 receptor agonist, lixisenatide, on the propagation of α-synuclein pathology. We found that lixisenatide reduced α-synuclein phosphorylation, aggregation, and propagation in cells treated with α-synuclein preformed fibrils, and that these effects were accompanied by decreased mitochondrial dysfunction and apoptosis. Additionally, lixisenatide treatment alleviated motor dysfunction and dopaminergic cell neurodegeneration 20 weeks after stereotactic injection of α-synuclein preformed fibrils into the striatum of wild-type mice. In addition, lixisenatide inhibited α-synuclein phosphorylation and seeding between neurons, mediated by neuronal lymphocyte-activation gene 3 expression. This study provides new insights into the mechanism underlying the disease-modifying effects of glucagon-like peptide-1 receptor agonists in the treatment of Parkinson’s disease.

## Graphical abstract

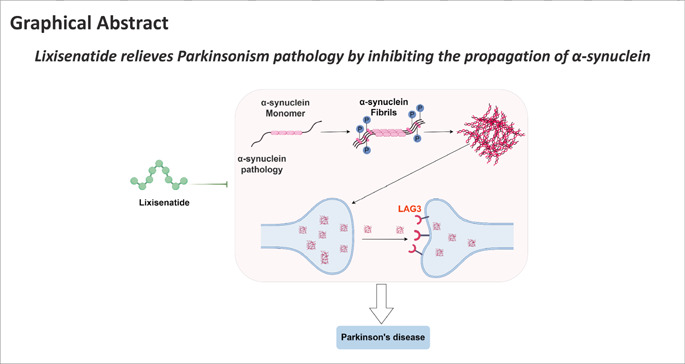

## Introduction

Parkinson’s disease (PD) is one of the most common neurodegenerative diseases, characterized by both motor and non-motor symptoms. The pathological hallmark of PD is abnormal accumulation and aggregation of α-synuclein (α-Syn) in Lewy bodies and selective degeneration of dopaminergic neurons in the substantia nigra (Fearnley and Lees, 1991). α-Syn can spread among neurons by self-amplification, propagation, and transmission (Volpicelli-Daley et al., 2011; Luk et al., 2012), and α-Syn aggregates spreading in the brain is associated with disease progression (Grosso Jasutkar et al., 2022). At present, PD treatment primarily focuses on relieving the clinical symptoms, but there is no effective medication available to halt progression of the disease. Clarifying PD pathogenesis and developing drugs that target α-Syn propagation will provide an effective disease-modifying treatment for PD.

Recent studies have demonstrated an association between type 2 diabetes mellitus (T2DM) and PD. T2DM is an established risk factor for PD and promotes its progression (Chohan et al., 2021). In addition, PD and T2DM share overlapping pathogenic mechanisms, including insulin resistance, amyloid aggression, chronic inflammation, oxidative stress, and mitochondrial dysfunction (Cheong et al., 2020; Sabari et al., 2023). Drugs initially developed for T2DM have shown positive results on PD in both preclinical and clinical trials, particularly glucagon-like peptide-1 (GLP-1) receptor agonists (Athauda et al., 2017; Meissner et al., 2024). GLP-1 is an endogenous peptide belonging to the incretin hormone family. Glucagon-like peptide-1 receptor (GLP-1R) is widely expressed in the pancreas, stomach, duodenum, lung, and brain. Researchers have observed increased GLP-1R mRNA expression in the brains of individuals with PD (Yun et al., 2018). GLP-1 binding to its receptor (GLP-1R) enhances insulin secretion, reduces glucagon release, and boosts muscle glucose uptake (Cheng et al., 2022; Nowell et al., 2023; Mariam and Niazi, 2024). Numerous clinical trials investigating the effects of GLP-1R agonists on PD have reported promising results. Specifically, treatment with exenatide or lixisenatide resulted in significant improvement in MDS Unified-Parkinson Disease Rating Scale scores. Additionally, liraglutide treatment has been associated with enhanced scores related to activities of daily living (Athauda et al., 2017; Kalinderi et al., 2024; Meissner et al., 2024). Notably, the neuroprotective effects of exenatide on PD persisted even after a 12-week washout period, suggesting the possibility of disease modification (Athauda et al., 2017). Furthermore, preclinical studies have demonstrated the neuroprotective effects of GLP-1R agonists on PD. Liraglutide and lixisenatide demonstrated neuroprotective properties, including preservation of tyrosine hydroxylase (TH)-positive neurons, a reduction in apoptosis, and amelioration of motor impairments in a 1-methyl-4-phenyl-1,2,3,6-tetrahydropyridine (MPTP)-induced mouse model of PD (Liu et al., 2015). Exendin-4 increased the number of both TH- and vesicular monoamine transporter 2–positive neurons in the substantia nigra and normalized dopamine imbalance in a 6-hydroxydopamineinduced animal model of PD (Bertilsson et al., 2008). NLY01, a long-acting GLP-1 analog, was found to directly prevent microglia-mediated conversion of astrocytes to the A1 phenotype, thus improving neuronal survival (Yun et al., 2018).

As mentioned above, α-Syn seeding and transmission between neurons are associated with PD progression. GLP-1R agonists have been shown to modify PD in clinical trials. However, little is known about the effects of GLP-1R agonists on α-Syn pathology. Furthermore, animal models of Parkinson’s disease induced by toxins fail to fully replicate the pathology associated with α-Syn. To elucidate the mechanism of GLP-1R agonists in PD disease modification, we studied effects of the candidate drug lixisenatide on α-Syn pathology. Lixisenatide is a well-tolerated, short-acting GLP-1R agonist that can cross the blood–brain barrier (BBB). Lixisenatide was shown to protect against PD in a phase II clinical trial (Meissner et al., 2024) and to have neuroprotective effects in an MPTP-induced animal model of PD (Liu et al., 2015; Pfeffer et al., 2015; Muskiet et al., 2018; Salameh et al., 2020; Terauchi et al., 2022). However, the mechanism by which lixisenatide protects against PD treatment remains unclear. Here, we observed the effects of lixisenatide on α-Syn pathology in *in vitro* and *in vivo* models established by α-Syn preformed fibrils (PFFs).

## Methods

### Cell culture

The HEK-293 cell line (RRID: CVCL_0045) and SH-SY5Y cell line (RRID: CVCL_0019) were obtained from the American Type Culture Collection. The HEK-293 cell line was used to generate a cell line stably expressing GFP-tagged α-Syn A53T (α-Syn-HEK-293 cells), as previously described (Meng et al., 2023). α-Syn-HEK-293 cells and SH-SY5Y cells were cultured independently in complete medium (Procell, Wuhan, China; Cat# CM-0001 for HEK-293 cells, Cat# CM-0208 for SH-SY5Y cells), and maintained in a humidified incubator with 5% CO_2_ at 37°C. Both of the cell lines were used as cell models of PD.

### Primary cortical neuron culture

Primary cortical neurons were prepared from the cortices of wild-type mice aged embryonic day 18 and cultured in neurobasal media (Gibco, Waltham, MA, USA, Cat# 21103049) supplemented with 2% B27 (Gibco, Cat# 17504044), 1% L-glutamine (Gibco, Cat# 35050-061), and 1% penicillin-streptomycin (Cat# BL505A, Biosharp, Hefei, China) in a 37°C, 5% CO_2_, humidified incubator. Half of the growth medium was replaced with fresh complete medium every 3 to 4 days. Mouse α-Syn PFFs (3 µg/mL) were added to the culture medium at 7 days *in vitro* (DIV 7). On DIV 14 the neurons were stained with propidium iodide (PI) and Hoechst and analyzed by immunofluorescence microscopy.

### Animals

Wild-type (C57BL/6) mice were purchased from the Shulaibao Biotechnology Co., Ltd (Wuhan, China, license No. SCXK (E) 2021-0025). Forty-eight male C57BL/6 mice (3 months old, approximately 25–30 g) were housed at four mice per cage. All mice were maintained in a specific-pathogen–free environment with a 12/12-hour light/dark cycle at a constant temperature (22 ± 2°C). Food and water were available *ad libitum*. At the age of 3 months, the mice were randomly divided into groups that received stereotactic injection (ST) of α-Syn PFFs or phosphate-buffered saline (PBS). One week later, the mice were injected intraperitoneally with lixisenatide (10 nmol/kg, MedChemexpress, Monmouth Junction, NJ, USA, Cat# HY-P0119) or an equal volume of sterile saline, and this was repeated daily for 20 weeks. The experimental groups were as follows: PBS + Vehicle (Veh) (PBS + saline), α-Syn PFFs + Veh (α-Syn PFFs + saline), PBS + Lix (PBS + lixisenatide), and α-Syn PFFs + Lix (α-Syn PFFs + lixisenatide). All experimental protocols were approved by Institutional Animal Care and Use Committee (IACUC) of Renmin Hospital of Wuhan University on January 24, 2024 (approval No. 20240106B).

### Preparation and transduction of α-synuclein preformed fibrils

α-Syn monomers were purified as described previously (Volpicelli-Daley et al., 2014). Dialyzed and lyophilized α-Syn monomers were resuspended in sterile PBS and centrifuged at 100,000 × *g* at 4°C for 20 minutes. The resulting α-Syn monomers were diluted to a final concentration of 1 mg/mL. Then, the mixture was stirred at 37°C and 1000 r/min for 7 days in a thermomixer (Eppendorf, Hamburg, Germany). The α-Syn PFFs were sonicated before using for further experiments.

### Thioflavin T aggregation detection

The kinetics of α-Syn fibrillation were monitored by thioflavin T (ThT) fluorescence at multiple time points. First, the α-Syn monomers were mixed with saline or lixisenatide (10 nM) to a final concentration of 1 mg/mL and incubated. At different time points, 10 µL samples were removed and incubated with 2 µL 1 mM ThT (Sigma-Aldrich, Darmstadt, Germany, Cat# T3516) diluted in 90 µL PBS for 5 minutes in the dark at 37°C. The fluorescence was detected at 450 nm excitation and 510 nm emission using a microplate reader (Spectra Max i3x, Molecular Devices, San Jose, CA, USA).

### Stereotactic injection

The mice were deeply anesthetized by inhalation of 2% isoflurane (RWD Life Science, Shenzhen, China, Cat# R510-22). Then, the mice received ST of PBS or α-Syn PFFs into the right striatum (coordinates: anteroposterior (AP) +0.2 mm, mediolateral (ML) –2.0 mm, and dorsoventral (DV) –2.7 mm) (Paxinos and Franklin, 2013). The total injection amount was 10 µL α-Syn PFFs (1 mg/mL) or 10 µL PBS at a speed of 0.2 µL/min. After completing the injection, the needle was left in place for 5 minutes before being withdrawn. Post-surgery, the mice were maintained at a warm temperature for 12 hours.

### Cell Counting Kit 8 assay

The Cell Counting Kit 8 (CCK-8) assay (Servicebio, Wuhan, China, Cat# G4103) was performed to assess cell viability. α-Syn-HEK-293 cells were cultured in a 96-well plate in a 37°C, 5% CO_2_, humidified incubator. α-Syn PFFs were transduced into the cells with or without lixisenatide (10 nM) for 48 hours, after which the medium was replaced with fresh Dulbecco’s Modified Eagle Medium (DMEM, Gibco, Cat# C11995500BT). CCK-8 solution (10 µL) was added to each well, and the plates were incubated for 1–3 hours. The data were collected using a microplate reader at an absorbance of 450 nm.

### Propidium iodide–Hoechst assay

The transduction system consisted of 9.6 µL Lipo8000 transfection reagent (Beyotime, Shanghai, China, Cat# C0533), 125 µL Opti-MEM Medium (Gibco, Cat# 31985070), and 6 µg α-Syn PFFs in each well. α-Syn PFFs were transduced into SH-SY5Y cells with or without lixisenatide (10 nM) treatment for 48 hours in a 6-well plate, after which the medium in each well was replaced with 1 mL cell staining buffer (Beyotime, Cat# C1056). Then, 5 µL of Hoechst 33342 staining solution and 5 µL of PI staining solution were added to each well, and the plate was incubated at 37°C for 20 minutes. Next, the staining buffer was removed, and the cells were rinsed twice with PBS. The experiment was repeated using neurons instead of SH-SY5Y cells. Fluorescence microscopy (Olympus, Tokyo, Japan) was used to observe the stained cells under wavelengths (E_x_ = 350 nm; E_m_ = 460 nm) for Hoechst 33342 or wavelengths (E_x_ = 535 nm; E_m_ = 617 nm) for PI respectively.

### Reactive oxygen species assay

Loaded DCFH-DA probe (Nanjing Jiancheng, Nanjing, China, Cat# E004-1-1) was added into SH-SY5Y cells at a final concentration of 10 µM, and the plate was incubated at 37°C for 30 minutes. Then, the complete medium (Procell, Cat# CM-0208) was removed, and the cells were washed with PBS twice. Fluorescence microscopy was performed at excitation wavelengths of 488 nm and emission wavelengths of 525 nm.

### Animal living computed tomography imaging

The mice were deeply anesthetized by inhalation of 2% isoflurane, and the fur was removed from their abdominal and cranial regions. Subsequently, fluorescein isothiocyanate (FITC)-lixisenatide (2.5 mM, QYAOBIO, Shanghai, China) or an equal volume of saline was administered by intraperitoneal injection. Photographic documentation was conducted at 30-minute intervals post-injection, over 120 minutes, utilizing a live-animal CT imaging system (NightOWL II LB 983 NC 100, BERTHOLD Technologies, Bad Wildbad Baden, Germany).

### Transmission electron microscopy

A droplet of α-Syn PFFs with or without lixisenatide was placed onto a copper grid for 1 minute. The copper grid was pretreated with the Glow Discharge Cleaning System (PELCO easiGlow, Redding, CA, USA) for 1 minute, ensuring even distribution of the subsequent sample staining. The sample was rinsed twice with 2% uranyl acetate (College of Life Sciences, Wuhan University, China) for 3 minutes before staining for 1 minute. Then the copper grid was allowed to dry for 10 minutes before observation. Images were captured by transmission electron microscopy (TEM) (JEM-1400plus, Tokyo, Japan). A 2% glutaraldehyde solution (Servicebio) was injected to anesthetized mice, and the striatal tissue was quickly isolated and cut into 1 mm^3^ pieces, then fixed at room temperature for 4 hours. The tissue was then loaded onto a copper grid for electron microscopy to assess mitochondrial damage by examining the outer membrane and overall structure.

### Proteinase K digestion

Proteinase K (PK; BioFroxx, Guangzhou, China) was added to α-Syn PFFs (with or without lixisenatide) at various doses (0, 0.5, 1, and 1.5 mg/mL), and the samples were incubated for 0.5 hours at 37°C. A protease inhibitor (Topscience, Shanghai, China, Cat# C0001) was used to stop the digestion process. The samples were boiled and stained with Coomassie blue (BioFroxx, Guangzhou, China, Cat# 1912GR025).

### Western blotting

Mouse brain tissue was lysed with RIPA buffer containing protease and phosphatase inhibitors, followed by sonicating and centrifuging at 10,605 × *g* for 20 minutes at 4°C. Protein concentrations were quantified by BCA assay. The proteins were separated on 10% bis-tris-sodium dodecyl sulfate (SDS)-PAGE gels. The proteins were then transferred to nitrocellulose membranes (Merck Millipore, Tullagreen, Carrigtwohill, Ireland, Cat# HATF00010), and the membranes were blocked with 5% skim milk in Tris-buffered saline (TBS) with 0.1% Tween-20 for 2 hours and incubated with the appropriate antibody overnight at 4°C. The primary antibodies used in this study were: anti-TH (rabbit mAb, 1:1000, Cell Signaling Technology, Danvers, MA, USA, Cat# 58844, RRID: AB_2744555), anti-α-Syn (p-Ser129) (rabbit mAb, 1:1000, Cell Signaling Technology, Cat# 23706), anti-GLP-1R (rabbit pAb, 1:500, Immunoway, Plano, TX, USA, Cat# YT1916), anti-LAG3 (rabbit pAb, 1:1000, Proteintech, Wuhan, China, Cat# 80867-1-RR, RRID: AB_2918917), and anti-β-actin (rabbit pAb, 1:3000, Signalway, Greenbelt, MD, USA, Cat# 48139). Subsequently, the membranes were washed three times for 10 minutes each and incubated with a horseradish peroxidase (HRP)-conjugated secondary antibody (goat anti-rabbit IgG H&L(HRP), 1:4000, Zenbio, Chengdu, China, Cat# 511203, RRID: AB_2927753) at room temperature for 1 hour. The signals were developed using an enhanced chemiluminescence reagent (Bio-Rad, Boulder, CO, USA, Cat# 1705061) and visualized using an imaging system (Bio-Rad).

### Extraction of soluble and insoluble fractions of cells and mouse striatal tissue

Mouse striatal tissue or α-Syn-HEK-293 cells were lysed in TBS (50 mM Tris, 150 mM NaCl, pH 7.6) containing protease inhibitors, phosphatase inhibitors (Topscience, Cat# C0003), and 1% Triton X-100 (TX-100) and incubated on ice for 30 minutes. The lysates were then ultrasonicated and centrifuged at 100,000 × *g* for 1 hour, and the supernatant containing the soluble fraction was collected. The pellet was washed with TBS containing 1% TX-100, ultrasonicated, and centrifuged at 100,000 × *g* for 30 minutes; the supernatant was discarded. The pellet was then suspended in TBS with 2% SDS and lysed on ice for 30 minutes, followed by ultrasonication and centrifugation at 100,000 × *g* for 1 hour to obtain the insoluble fraction. Protein concentration was determined using a BCA protein assay kit (Thermo Fisher, Richardson, TX, USA, Cat# 23225).

### Intraperitoneal glucose tolerance test and intraperitoneal insulin tolerance test

Mice were subjected to a 12-hour fasting period before intraperitoneal administration of 20% glucose to measure glucose tolerance (intraperitoneal glucose tolerance test [IPGTT]). One week later, an intraperitoneal insulin tolerance test (IPITT test) was conducted to measure insulin tolerance. The mice were fasted for 4 hours before receiving 0.75 U/kg insulin (10 mL:400 U) intraperitoneally. Mice were allowed *ad libitum* access to water during the IPGTT and IPITT tests. Peripheral blood samples were collected from the tail vein at 0, 30, 60, and 90 minutes to measure glucose levels (OneTouch Verio Vue, North Brunswick, NJ, USA).

### Enzyme-linked immunosorbent assay

Insulin (Cat# MU30432), interleukin (IL)-6 (Cat# MU30044), IL-1β (Cat# MU30369), and tumor necrosis factor-α (TNF-α) (Cat# MU30030) enzyme-linked immunosorbent assay (ELISA) kits were obtained from Bioswamp, Wuhan, China. The serum insulin concentrations and the levels of IL-6, IL-1β, and TNF-α in the striatum were assessed using the ELISA kits, following the manufacturer’s instructions.

### Hematoxylin and eosin staining

Peripheral mouse organs, including the spleen, stomach, pancreas, liver, kidney, and colon, were fixed in 4% paraformaldehyde (Servicebio, Cat# G1101). The fixed tissues were then dehydrated, paraffin-embedded, and sliced into 4 µm-thick sections. Following dewaxing and rehydration, the sections were placed hematoxylin and eosin high-definition constant staining pretreatment solution for 1 minute. Then, the sections were stained with hematoxylin solution (Servicebio, Cat# G1076) for 3 minutes, washed with water for 1 minute, and placed in hematoxylin differentiation solution. After washing for 20 seconds, the slices were dyed with eosin Y dye solution (Servicebio, Cat# G1076). The sections were then dehydrated completely before being sealed with neutral gum. The sections were observed under a microscope (Olympus), and images were acquired using a Digital Pathology System (Panoramic MIDI 3DHISTECH, Budapest, Hungary).

### Immunohistochemistry

Brain tissue sections were dewaxed with xylene and rehydrated in ethanol, antigen repair was performed, and endogenous peroxidase was blocked. Then the sections were blocked with 3% BSA for 1 hour and incubated with primary antibody––anti-TH (rabbit mAb, 1:1000, Cell Signaling Technology, Cat# 58844, RRID: AB_2744555) or anti-α-Syn (p-Ser129) (mouse mAb, 1:1000, BioLegend, San Diego, CA, USA, Cat# 825701)––at 4°C overnight. Next, the slides were incubated with secondary antibody (HRP-labeled goat anti-rabbit IgG, ZSGB-BIO, Beijing, China, Cat# PV-6001, RRID: AB_2864333 and HRP-labeled goat anti-mouse IgG, ZSGB-BIO, Cat# PV-6002, RRID: AB_2864334) at 37°C for 20 minutes. Signals were developed by DAB staining (ZSGB-BIO, Cat# ZLI-9017). TH-positive neurons were quantified as described previously (Zhou et al., 2023). For immunofluorescence, α-Syn-HEK-293 cells were incubated with anti-α-Syn (p-Ser129) (mouse mAb, 1:500, BioLegend, Cat# 825701, RRID: AB_2564891), SH-SY5Y cells were incubated with anti-Cytochrome c oxidase (COX) IV (rabbit pAb, 1:600, Abcam, Trumpington, Cambridge, UK, Cat# ab16056, RRID: AB_443304), neurons were incubated with anti-COX IV (rabbit pAb, 1:600) and anti-microtubule-associated protein 2 (MAP2) (mouse mAb, 1:200, Proteintech, Cat# 67015-1-Ig, RRID: AB_2882331), and brain tissue sections were incubated with primary antibodies (including anti-GLP-1R [rabbit, pAb, 1:200, Immunoway, Cat# YT1916], anti-TH [mouse mAb, 1:1000, Santa Cruz Biotechnology, Dallas, TX, USA, Cat# sc-25269, RRID: AB_628422], anti-LAG3 [rabbit, pAb, 1:200, Proteintech, Cat# 80867-1-RR, RRID: AB_2918917], anti-α-Syn (p-Ser129) [1:500, Cat# 825701], and anti-ionized calcium binding adapter molecule 1 (IBA1) [rabbit, pAb, 1:1000, Wako, Chuo-Ku, Osaka, Japan, Cat# 019-19741, RRID: AB_839504]) for 12 hours at 4°C. The cells and tissue sections were then incubated with secondary antibody (including Alexa Fluor 488 donkey anti-rabbit IgG (H+L) [1:500, Antgene, Wuhan, China, Cat# ANT024, RRID: AB_3107111], Alexa Fluor 488 donkey anti-mouse IgG (H+L) [1:500, Antgene, Cat# ANT023], Alexa Fluor 594 donkey anti-rabbit IgG (H+L) [1:500, Antgene, Cat# ANT030, RRID: AB_3107110], and Alexa Fluor 594 donkey anti-mouse IgG (H+L) [1:500, Antgene, Cat# ANT029]) at room temperature for 1 hour, stained with 4′,6-diamidino-2-phenylindole (DAPI) (1:1000, Abcam, Cat# ab228549), and sealed with antifade mounting medium. Immunohistochemistry (IHC) images were captured using a microscope (Olympus or Leica TCS SP8, Wetzlar, Germany), and a Digital Pathology System (Panoramic MIDI 3DHISTECH). Images were analyzed using ImageJ 1.51 j8 (National Institutes of Health, Bethesda, MD, USA).

### Behavioral tests

Mice were subjected to behavioral assessments 20 weeks after PFFs ST. The rotarod test was used to evaluate the extent of motor dysfunction, while the grid test, grip strength test, and pole test were used to assess neuromuscular coordination and strength.

#### Rotarod test

Mice were placed vertically relative to the rod. The rate of the rod was gradually increased from 5 revolutions/min to 40 revolutions/min within 3 minutes. The mice were trained three times a day for 3 consecutive days before the formal test was carried out. The mice were allowed to rest between each trial during training. The time that the mice took to fall from the rod was recorded.

#### Grid test

Mice were placed on the wire grid (1.5 m by 1.5 m with apertures of 5 mm in diameter) and allowed to adapt for 30 seconds until their claws were tightly wrapped around the wire mesh. Then, the grid was turned over, and the time it took the mice to fall was recorded.

#### Grip strength test

Mice were placed on a wire for 2 minutes to attain stability. Then, the mouse’s tail was pulled, guiding its forelimbs and hindlimbs to quickly pass through the wire mesh of the pressure receptor three consecutive times. Grip force was averaged.

#### Pole test

The mice were placed at the top of a rough-surfaced wooden pole measuring 45 cm in length and 1 cm in diameter. The time it took the mouse to descend halfway and then all the way was recorded. Training took place twice before the formal test.

### Statistical analysis

Sample sizes were not calculated, but the group numbers are similar to those reported in prior studies (Pan et al., 2022; Dai et al., 2023). The data were analyzed using GraphPad Prism10 (GraphPad, San Diego, CA, USA, www.graphpad.com). Relative protein expression levels were normalized to β-actin for the western blotting analysis. The Shapiro–Wilk normality test was utilized to determine the normality of the data distribution. Once the normality of the data distribution and homogeneity of variances were confirmed, two-tailed unpaired Student’s *t*-test was utilized to analyze the differences between the two groups of samples, and one-way analysis of variance followed by Tukey’s multiple comparisons correction was performed for comparison among multiple groups. One-way analysis of variance was applied to data analysis, log transformation or nonparametric test (Kruskal–Wallis test) was utilized for non-normally distributed data, and *P* < 0.05 was considered statistically significant.

## Results

### Lixisenatide reduces α-synuclein aggregation and phosphorylation *in vitro*

**Figure 1:**
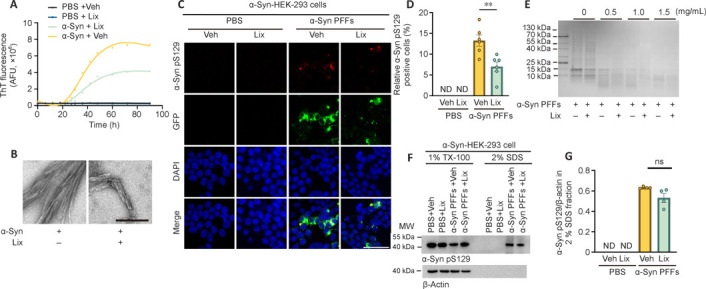
Lixisenatide inhibited α-Syn aggregation and phosphorylation *in vitro*. (A) The aggregation kinetics of α-Syn (1 mg/mL) with or without lixisenatide (10 nM) as detected by ThT fluorescence. (B) Electron microscopy images of α-Syn fibrils formed by monomeric α-Syn with or without lixisenatide (scale bar: 200 nm). (C) α-Syn aggregation (green) and pS129 (red) immunofluorescence signals in α-Syn-HEK-293 cells, which were transduced with α-Syn preformed fibrils with or without lixisenatide treatment (scale bar: 50 µm). (D) Quantification of the relative percentage of α-Syn pS129-positive cells. (E) Proteinase K digestion patterns of α-Syn fibrils formed by monomeric α-Syn with or without lixisenatide. (F) The levels of α-Syn pS129 in the TX-100-soluble and SDS-insoluble fraction. (G) Quantification of α-Syn pS129 levels in the SDS-insoluble fraction (*n* = 4 independent experiments). Data were analyzed by unpaired Student’s *t*-tests (D, G). All data are shown as mean ± SEM, **P < 0.01. PBS + Veh: PBS + Saline; PBS + Lix: PBS + lixisenatide; α-Syn + Veh: α-Syn PFFs + Saline; α-Syn + Lix: α-Syn PFFs + lixisenatide. AFU: Arbitrary fluorescence unit; MW: molecular weight; ND: not detected; ns: not significant; ThT: Thioflavin T; α-Syn pS129: α-synuclein phosphorylation at the residue S129.

To explore whether lixisenatide affects α-Syn PFF aggregation, we incubated monomeric α-Syn with lixisenatide (10 nM) or saline *in vitro*. The aggregation kinetics was assessed by ThT fluorescence assay. We found that monomeric α-Syn aggregated and reached a plateau 60 hours after incubation at 37°C, while lixisenatide treatment slowed down the aggregation speed of α-Syn PFFs (**Figure 1A**). Then, TEM was used to explore the morphological characteristics of the aggregates. The fibrils in the α-Syn + Lix group were shorter and sparser than the fibrils in the α-Syn + Veh group (**Figure 1B**).

To measure the seeding properties of α-Syn, HEK-293 cells stably expressing GFP-tagged-α-Syn A53T were used as reporter cells (Meng et al., 2023). α-Syn PFFs treatment induced α-Syn aggregation and phosphorylation at the residue S129 (pS129), as previously reported (Oueslati, 2016; Meng et al., 2023). However, lixisenatide treatment alleviated the α-Syn aggregation and phosphorylation induced by α-Syn PFFs (**Figure 1C** and **D**). Next, we performed PK digestion and sequential extraction to elucidate the chemical properties of α-Syn. The PK digestion test showed that the α-Syn in the α-Syn PFFs + Lix group was more easily digested (**Figure 1E**). In sequential extraction experiments performed on α-Syn-HEK-293 cells, the fraction that was insoluble to 2% SDS in the α-Syn PFFs + Lix group showed a downward trend in pS129 levels compared with that in the α-Syn PFFs + Veh group, although the difference was not significant (**Figure 1F** and **G**). Collectively, these findings suggest that lixisenatide can reduce α-Syn aggregation and phosphorylation *in vitro*.

### Lixisenatide reduces the cell apoptosis and mitochondrial dysfunction induced by α-synuclein preformed fibrils

**Figure 2:**
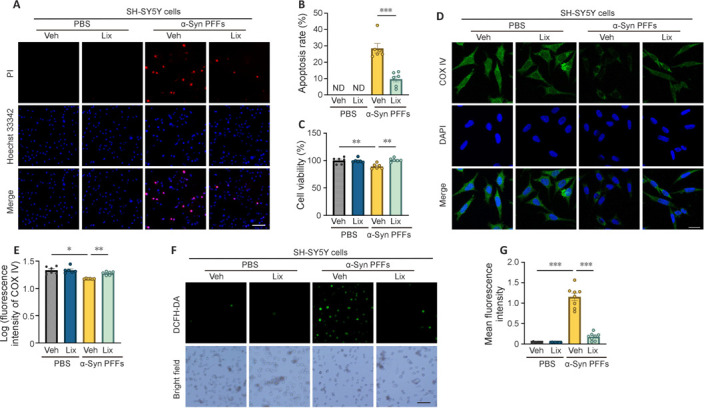
Lixisenatide relieved cell apoptosis and mitochondrial dysfunction *in vitro*. SH-SY5Y cells were treated with α-Syn PFFs in the absence or presence of lixisenatide for 48 hours. (A) PI (Red) and Hoechst 33342 (blue) double staining in SH-SY5Y cells (scale bar: 100 µm). (B) Apoptosis rate calculated by percentage of PI-positive cells in total cells (*n* = 6 independent experiments). (C) Cell viability was assessed by CCK-8 assay in α-Syn-HEK-293 cells (*n* = 6 independent experiments). (D) COX IV (green) were detected by immunofluorescence (scale bar, 25 µm). (E) Quantification of COX IV fluorescence intensity (*n* = 6 independent experiments), and the data were presented with a logarithmic transformation. (F) DCFH-DA staining (green) was used to detect the level of ROS (upper panels), bright view of the cells (lower panels) (scale bar: 100 µm). (G) Quantification of DCFH-DA fluorescence intensity (*n* = 8 independent experiments). Data were analyzed by unpaired Student’s *t*-tests (B), and one-way analysis of variance followed by Tukey’s multiple comparisons correction (C, E, G). All data are shown as mean ± SEM, *P < 0.05, **P < 0.01, ***P < 0.001. CCK-8: Cell Counting Kit 8; Lix: lixisenatide; ND: not detected; PI: propidium iodide; ROS: reactive oxygen species; α-Syn PFFs: α-synuclein preformed fibrils.

Cell apoptosis and mitochondrial dysfunction are the main pathological changes that occur in the PD cell model. We conducted PI-Hoechst and CCK-8 assays to investigate the effect of lixisenatide on cell apoptosis and viability. The apoptosis rate of SH-SY5Y cells in the α-Syn PFFs + Lix group was significantly lower than that in the α-Syn PFFs + Veh group (*P* < 0.001), which suggested that lixisenatide treatment significantly reduced the apoptosis induced by α-Syn PFFs (**Figure 2A** and **B**). A similar protective effect was observed in primary neurons cultured from wild-type mouse cortical tissue (**Additional Figure 1A** and **B**). In α-Syn-HEK-293 cells, lixisenatide partially restored the impairment in cell viability induced by α-Syn PFFs (**Figure 2C**). Mitochondrial dysfunction is closely related to degeneration of dopaminergic neurons (Henrich et al., 2023). Therefore, to evaluate mitochondrial function, we assessed COX IV expression both in SH-SY5Y cells and primary neurons. COX IV, a multi-subunit enzyme complex located in the mitochondrial inner membrane, is a biomarker of mitochondria. Immunofluorescence staining revealed that COX IV expression was decreased in the α-Syn PFFs + Veh group, which indicated that mitochondrial dysfunction was induced by α-Syn PFFs. However, lixisenatide treatment partially mitigated the reduction in COX IV expression in SH-SY5Y cells, as seen in the α-Syn PFFs + Lix group (*P* = 0.003; **Figure 2D** and **E**). COX IV was also better retained in neurons treated with lixisenatide (**Additional Figure 1C** and **D**). These results indicate that lixisenatide can protect cells from mitochondrial dysfunction. Furthermore, we assessed ROS levels to gauge oxidative stress. The results showed that the levels of oxidative stress in the α-Syn PFFs + Lix group were lower than those in the α-Syn PFFs + Veh group (*P* < 0.001; **Figure 2F** and **G**). Collectively, these findings suggest that lixisenatide may reduce apoptosis and maintain mitochondrial energy homeostasis.

### Lixisenatide improves motor dysfunction in an α-synuclein preformed fibrils–induced mouse model of Parkinson’s disease

**Figure 3:**
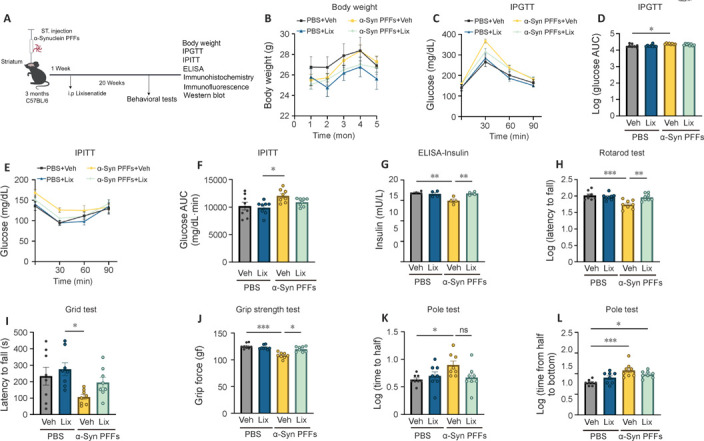
Lixisenatide improved metabolic dysfunction and motor function. (A) Schematic diagram of the experimental design. (B) Body weight of the mice (*n* = 8 mice per group). (C, D) IPGTT was performed to measure glucose tolerance. The mice were administrated intraperitoneally with 20% glucose. Baseline fasted glucose concentration (C) and AUC of IPGTT (D) (*n* = 8 mice per group). 1 mM = 18 mg/dL. (E, F) IPITT was performed to measure insulin tolerance of the mice. Baseline fasted glucose concentration (E) and AUC of IPITT (F) (*n* = 8 mice per group). (G) ELISA analysis of serum insulin level (*n* = 4 mice per group). (H–L) Rotarod test, grid test, grip strength test and pole test were performed (*n* = 8 mice per group). The data were presented using a logarithmic transformation (D, H, K, L). Data were analyzed using one-way analysis of variance followed by Tukey’s multiple comparisons correction (D, F–I, K, L) or Kruskal-Wallis test followed by Dunnett’s T3 multiple comparisons test (J). All data are shown as mean ± SEM, *P < 0.05, **P < 0.01, ***P < 0.001. AUC: Area under curve; ELISA: enzyme-linked immunosorbent assay; IPGTT: Intraperitoneal glucose tolerance test; IPITT: Intraperitoneal insulin tolerance test; Lix: lixisenatide; ns: not significant; Veh: saline; α-Syn PFFs: α-synuclein preformed fibrils.

To investigate the effects of lixisenatide on PD, we delivered α-Syn PFFs into the striatum of 3-month-old wild-type mice by ST, starting one week later, and intraperitoneally injected the mice with lixisenatide (10 nmol/kg per day) for 20 weeks (**Figure 3A**). The α-Syn PFFs–induced mice model is an established model of idiopathic synucleinopathy (Uemura et al., 2021). First, we assessed the effect of lixisenatide on metabolism. During the course of lixisenatide treatment, we systematically monitored the body weight of the mice and observed that administration of lixisenatide at a dosage of 10 nmol/kg/d did not significantly affect their body weight (**Figure 3B**). Subsequently, we assessed glucose tolerance in the mice using the IPGTT (**Figure 3C** and **D**) and evaluated insulin tolerance by the IPITT (**Figure 3E** and **F**). We observed that glucose levels reached their peak value at 30 minutes and returned to baseline within 90 minutes following IPGTT. The IPGTT results revealed impaired glucose clearance in the α-Syn PFFs + Veh group compared with the PBS + Veh group (*P* = 0.011). Conversely, the α-Syn PFFs + Lix group exhibited a reduction in blood glucose levels, indicating enhanced glucose tolerance compared with the α-Syn PFFs + Veh group (**Figure 3C** and **D**). The degree of blood glucose reduction in response to insulin injections serves as an indicator of insulin sensitivity (Campbell and Newgard, 2021). Notably, elevated glucose levels were observed 30 minutes post-insulin injection in the α-Syn PFFs + Veh group compared with the other groups (**Figure 3E**). Area under curve (AUC) analysis indicated impaired insulin tolerance in the α-Syn PFFs group compared with the PBS + Veh group and the PBS + Lix group (*P* = 0.017; **Figure 3F**). Although a reduction in glucose levels was observed in the α-Syn PFFs + Lix group, suggesting improved insulin sensitivity, this reduction did not reach statistical significance (**Figure 3F**). In addition, we found that α-Syn PFFs treatment decreased peripheral insulin secretion (–12.3%, *P* = 0.003), while lixisenatide treatment increased insulin secretion (11.1%, *P* = 0.007; **Figure 3G**). Subsequently, we assessed the motor function of the mice using a series of tests, including the rotarod test, grid test, grip strength test, and pole test. In the rotarod test, the mice in the α-Syn PFFs + Lix group showed a significantly longer latency to fall compared with the mice in α-Syn PFFs + Veh group (*P* = 0.005; **Figure 3H**). However, in the grid test there was no significant difference between the α-Syn PFFs + Lix group and α-Syn PFFs + Veh group (**Figure 3I**). The grip strength test revealed enhanced hind limb muscle strength in the α-Syn PFFs + Lix group relative to the mice in α-Syn PFFs + Veh group (*P* = 0.043; **Figure 3J**). In the pole test, the α-Syn PFFs + Lix group exhibited an decrease in the latency of descent compared with the α-Syn PFFs + Veh group (**Figure 3K** and **L**), although this difference was not significant. These findings suggest that lixisenatide has a protective impact on insulin resistance and motor function in a mouse model of PD.

### Lixisenatide alleviates Parkinson’s disease-related pathology in an α-synuclein mouse model

**Figure 4:**
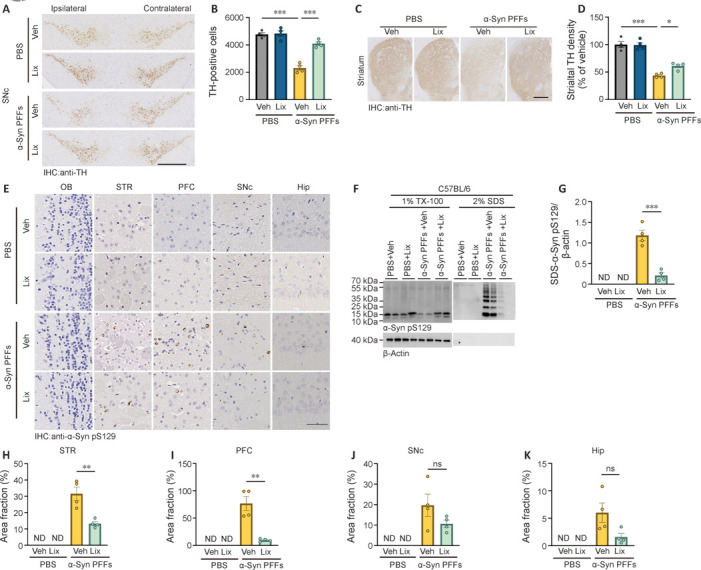
Lixisenatide resecured TH neurons and reduced parkinsonism-like pathology in α-Syn PFFs induced mice. (A) IHC staining of TH-positive cells in SNc (scale bar: 625 µm). (B) Quantification of the ipsilateral TH-positive neurons in SNc (*n* = 4 mice per group). (C) IHC staining of TH in striatum (scale bar: 1.25 mm). (D) Quantification of the ipsilateral TH-positive density in striatum (*n* = 4 mice per group). (E) IHC detection of α-Syn pS129 in the OB, STR, PFC, SNc, and Hip (scale bar: 50 µm). (F) Representative image of the western blot analysis of α-Syn pS129 in the TX-100-soluble and SDS-insoluble fraction in the striatum. (G) Quantification of α-Syn pS129 expression. Quantification of positive α-Syn pS129 inclusions in STR (H), PFC (I), SNc (J), Hip (K) (*n* = 4 mice per group, each brain with 3 slices). Data were analyzed using unpaired Student’s *t*-tests (G–K) or one-way ANOVA followed by Tukey’s multiple comparisons correction (B, D). All data are shown as mean ± SEM, *P < 0.05, **P < 0.01, ***P < 0.001. Hip: Hippocampus; Lix: lixisenatide; MW: molecular weight; ns: not significant; OB: olfactory bulb; PFC: prefrontal cortex; SNc: substantia nigra pars compacta; STR: striatum; TH: tyrosine hydroxylase; TX-100: Triton X-100; Veh: saline; α-Syn PFFs: α-synuclein preformed fibrils.

To verify the effect of lixisenatide on α-Syn pathology *in vivo*, we performed IHC analysis of brain tissue from the α-Syn PFFs–induced mouse model of PD. As reported previously, intrastriatal injection of α-Syn PFFs reduced the number of TH-positive neurons within the substantia nigra and striatum (Dai et al., 2023). Treatment with lixisenatide attenuated this effect, as seen in the α-Syn PFFs + Lix group (**Figure 4A–D**). Next, we detected α-Syn pS129-positive inclusions, a known characteristic of α-Syn pathology in the mouse brain. pS129 signals were detected in the striatum, prefrontal cortex, substantia nigra, and hippocampus 20 weeks after intrastriatal injection of α-Syn PFFs, but no signals were detected in the olfactory bulb, which is consist with a previous study (Yuan et al., 2022). Moreover, lixisenatide treatment ameliorated α-Syn PFFs –induced α-Syn pathology in the mouse brain. Notably, there was a significant reduction in pS129 signals in the striatum (–81.8%, *P* = 0.005) and prefrontal cortex (–156%, *P* = 0.002; **Figure 4E** and **H–K**) in the α-Syn PFFs + Lix group compared with the α-Syn PFFs + Veh group. Sequential extraction experiments revealed a reduction in α-Syn pS129 levels within the 1% TX-100 insoluble fraction in the α-Syn PFFs + Lix group compared with the α-Syn PFFs + Veh group (*P* < 0.001; **Figure 4F** and **G**). These findings suggest that lixisenatide may inhibit α-Syn aggregation and pathology and prevent the loss of dopaminergic neurons in the α-Syn PFFs–induced PD mouse model.

### Lixisenatide affects the spread of α-synuclein aggregates

**Figure 5:**
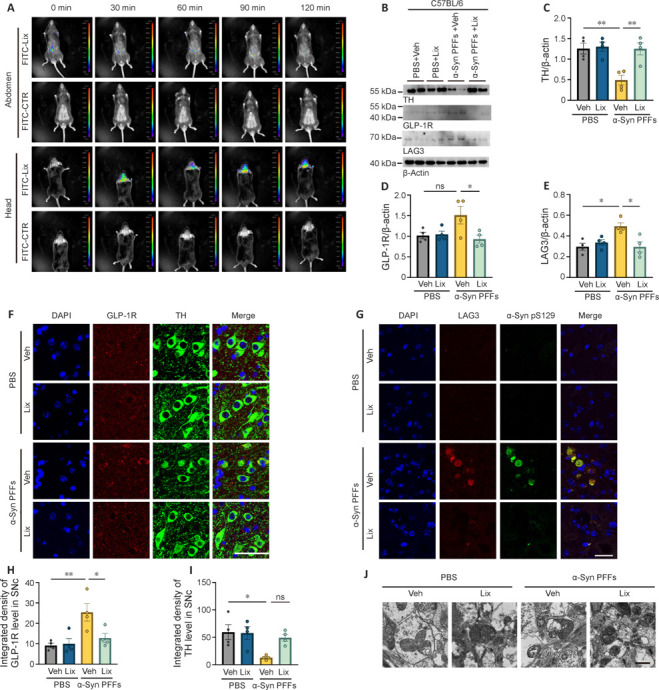
The neuroprotective mechanism of lixisenatide on α-Syn PFFs pathology. (A) Animal-living CT imaging, FITC-Lix (green) can be detected in abdominal cavity and brain of the mice within 120 minutes after FITC-lixisenatide intraperitoneal injection. (B) Representative image of the western blot analysis of TH, GLP-1R, LAG3 in the striatum of mice. (C–E) Quantification of levels of TH (C), GLP-1R (D), and LAG3 (E) (*n* = 4 mice per group). (F) Immunofluorescence staining of GLP-1R (red) and TH (green) in SNc (scale bar: 50 µm). (G) Immunofluorescence revealed co-localization of LAG3 (red) and α-Syn pS129 (green) in striatum (scale bar: 25 µm). (H, I) Quantification of GLP-1R and TH intensity (*n* = 4 mice per group). (J) Morphology assessment of mitochondria from the striatum by transmission electron microscopy (TEM) (scale bar: 0.5 µm). Data were analyzed by one-way analysis of variance with Tukey’s multiple comparisons correction (C, D, E, H, I). All data are shown as mean ± SEM. *P < 0.05, **P < 0.01. FITC-Lix: FITC-lixisenatide; GLP-1R: glucagon-like peptide-1 receptor; LAG3: lymphocyte-activation gene 3; Lix: lixisenatide; MW: molecular weight; ns: not significant; TH: tyrosine hydroxylase; Veh: saline; α-Syn PFFs: α-synuclein preformed fibrils; α-Syn pS129: α-synuclein phosphorylation at the residue S129.

The BBB permeability of a drug is a critical factor in evaluating its potential as a therapeutic option for central nervous system diseases (Wu et al., 2023). Thus, we ascertained the ability of lixisenatide to penetrate the BBB, employing small animal imaging techniques with fluorescent markers in murine subjects. The mice were injected intraperitoneally with FITC-lixisenatide. After injection, FITC-lixisenatide fluorescence was observed in the abdominal cavity in the experimental group, while the control group showed no fluorescence. FITC-lixisenatide fluorescence was observed in the mouse brain from 30 minutes to 120 minutes after injection (**Figure 5A**), proving that lixisenatide can cross the BBB. Additionally, HE staining of paraffin-embedded sections from different mouse organs showed no toxic effects of lixisenatide on the peripheral organs and tissues (**Additional Figure 2B**). As expected, lixisenatide, an GLP-1R agonist that is a promising drug for PD, can cross the BBB and is safe for peripheral organs.

α-Syn pathology is propagated by cell-to-cell transmission. Recent studies have reported that LAG3 plays a significant role in the intercellular transmission of α-Syn fibrils (Zhang et al., 2023). Western blot analysis was used to assess striatal TH, GLP-1R, and LAG3 expression levels in mice. The results revealed lower TH expression (–87.4%, *P* = 0.006), higher GLP-1R expression (47.8%, *P* = 0.035), and higher LAG3 expression (50.1%, *P* = 0.012) in the α-Syn PFFs + Veh group compared with the α-Syn PFFs + Lix group (**Figure 5B–E**). Immunofluorescence co-staining for TH and GLP-1R revealed upregulation of GLP-1R expression in the substantia nigra compacta in the α-Syn PFFs + Veh group, while treatment with lixisenatide alleviated these changes (**Figure 5F**, **H**, and **I**). Immunofluorescence analysis demonstrated colocalization of LAG3 and pS129 α-Syn within the striatal neurons. Notably, greater colocalization of LAG3 and pS129 was observed in the α-Syn PFFs + Veh group compared with the other groups, reinforcing the correlation between LAG3 and pS129 α-Syn (**Figure 5G**). Moreover, electron microscopy analysis of the mouse striatum revealed compromised mitochondrial outer membranes and cristae in the α-Syn PFFs + Veh group, whereas the mice in the α-Syn PFFs + Lix group exhibited more normal mitochondrial morphology (**Figure 5J**). Thus, LAG3 may be an important factor mediating the neuroprotective effects of lixisenatide on α-Syn pathology.

### Lixisenatide reduces inflammation

**Figure 6:**
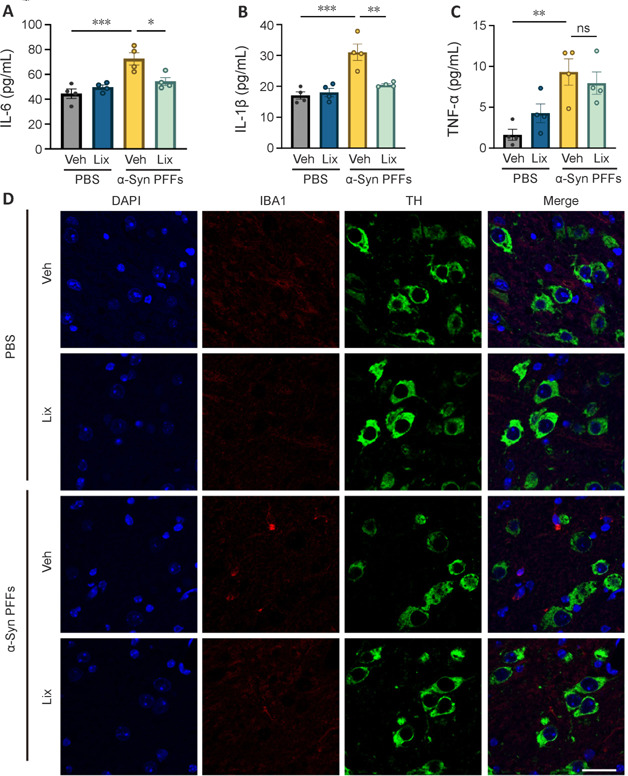
Lixisenatide reduced inflammation in α-Syn PFFs induced mice. (A–C) Enzyme-linked immunosorbent assay results of the levels of IL-6 (A), IL-1β (B), and TNF-ɑ (C) from the striatum of the mice (*n* = 4 mice per group). (D) Immunofluorescence staining of TH (Green) and IBA1 (Red) positive cells in SNc (scale bar: 25 µm). Data were analyzed using one-way analysis of variance with Tukey’s multiple comparisons correction (A–C). All data are shown as mean ± SEM. *P < 0.05, **P < 0.01, ***P < 0.001. IL: Interleukin; Lix: lixisenatide; ns: not significant; TH: tyrosine hydroxylase; TNF-α: tumor necrosis factor-α; Veh: saline; α-Syn PFFs: α-synuclein preformed fibrils.

One study demonstrated that inflammation and immune dysfunction play an essential role in PD pathogenesis (Tansey et al., 2022). To evaluate whether lixisenatide has an impact on inflammation in mice, we quantified inflammatory markers in the mouse striatum. We found that treatment with α-Syn PFFs elevated neuroinflammation in the brain, as indicated by increased levels of IL-6 (48%, *P* < 0.001), IL-1β (58.2%, *P* < 0.001), and TNF-α (140.4%, *P* = 0.004) in the α-Syn PFFs + Veh group compared with the PBS + Veh group. Conversely, lixisenatide treatment reduced the levels of IL-6 (–28.7%, *P* = 0.018) and IL-1β (–40.8%, *P* = 0.003) expression induced by α-Syn PFFs (**Figure 6A–C**). Furthermore, lixisenatide treatment decreased the number of IBA1-positive cells in the substantia nigra, indicating that lixisenatide inhibited neuroinflammation (**Figure 6D**). These results suggest that lixisenatide mitigates inflammation.

## Discussion

In recent years, the effects of GLP-1R agonists on PD treatment have been extensively studied in clinical trials and animal models of PD, and GLP-1R agonists have shown both neuroprotective and disease-modifying effects. Most preclinical studies have been performed in toxin-induced murine models, such as those induced by MPTP and 6-hydroxydopamine, which do not show α-Syn pathology. In our study, we explored the neuroprotective effects of lixisenatide on α-Syn pathology, using α-Syn PFFs–induced cellular and mouse models of PD. The findings demonstrate that lixisenatide inhibited α-Syn aggregation and propagation and mitigated cell death both *in vitro* and *in vivo*. This indicates that lixisenatide may represent an efficacious therapeutic option for PD disease modification.

GLP-1 is a hormone released by the gut. Previous studies have found that GLP-1R agonists exhibit protective effects in stroke, neurogenesis, neurodegeneration, and retinal repair through direct and indirect action on aspects of the central nervous system related to the gut–brain axis and insulinotropic effects or activate neurotrophic pathway (Grieco et al., 2019). A recent study investigated the ability of GLP-1R agonists to cross the BBB; the results indicated that exendin, lixisenatide, peptide 17, DA3-CH, and DA4-JC exhibit blood-to-brain permeability (Salameh et al., 2020). Conversely, liraglutide, semaglutide, and peptide 19 did not demonstrate measurable permeability through the BBB (Salameh et al., 2020). Compared with liraglutide and exenatide, lixisenatide and semaglutide exhibited reduced efficacy in activating Gi/o proteins and altered potency in receptor trafficking or subcellular localization of G protein–coupled receptors, and were associated with a lower incidence of pancreatitis (Wright et al., 2023). Lixisenatide is favored over other GLP-1R agonists for clinical applications due to its reduced side effect profile, high tolerability, and capacity to traverse the BBB (Pfeffer et al., 2015; Muskiet et al., 2018; Barrientos-Pérez et al., 2022; Terauchi et al., 2022). Moreover, lixisenatide showed a protective effect against PD in a recent phase II clinical trial (Kalinderi et al., 2024). Therefore, we selected lixisenatide as a candidate drug to investigate whether GLP-1R agonists influence α-Syn aggregation and transmission. To determine the direct or indirect effects of lixisenatide on PD, we utilized an *in vivo* imaging system to assess the BBB permeability of lixisenatide. Our results were consistent with previous reports and provided evidence for a direct effect of lixisenatide on the central nervous system (Salameh et al., 2020; Kalinderi et al., 2024).

Oxidative stress, neuroinflammation, mitochondrial dysfunction, and α-Syn aggregation and seeding are the characteristic features of PD pathogenesis (Morris et al., 2024). Oxidative stress facilitates α-Syn aggregation in a mouse model of PD (Scudamore and Ciossek, 2018). α-Syn aggregation increases mitochondrial oxidant stress, leading to dopaminergic neuronal death (Dryanovski et al., 2013). Moreover, mitochondria regulate ROS homeostasis and cell apoptosis (Melo et al., 2018). We found that treatment with α-Syn PFFs decreased COX IV expression and increased ROS production in SH-SY5Y cells and primary neurons. Lixisenatide treatment reduced oxidative stress, regulated mitochondrial function, and prevented the cell death caused by α-Syn PFFs. Our findings are consistent with previous studies showing that GLP-1R agonists can rescue oxidative stress, mitochondrial dysfunction and cell death in toxin-induced PD models (Lin et al., 2021; Wang et al., 2024). Consequently, the regulation of mitochondrial function by lixisenatide underscores its potential as a promising therapeutic agent for PD.

α-Syn aggregation and propagation are critical factors in PD progression (Borghammer, 2018; Mehra et al., 2019; Gatica-Garcia et al., 2024; Yang and Zhang, 2024). Various post-translational modifications have been shown to modulate α-Syn function and facilitate its aggregation (Manzanza et al., 2021; Gadhavi et al., 2022). Over 90% of synucleinopathy-affected brains exhibit pS129 expression (Fujiwara et al., 2002; Tarutani and Hasegawa, 2024). Consequently, pS129 is extensively utilized as a biomarker of α-Syn pathology (Gadhavi et al., 2022; Tarutani and Hasegawa, 2024). Cell-to-cell spread of α-Syn is crucial for the dissemination, severity, and onset of α-Syn toxicity (Wong and Krainc, 2017). However, little is known about whether GLP-1R agonists inhibit α-Syn aggregation and seeding. A previous study found that the GLP-1R agonist NLY01 protects against dopaminergic neurodegeneration and behavioral deficits in an α-Syn PFFs induced mouse model. NLY01 inhibited the microglia-mediated conversion of astrocytes into the neurotoxic reactive form, resulting in decreased α-Syn aggregation (Yun et al., 2018). In this study, we validated the efficacy of lixisenatide in inhibiting α-Syn aggregation and seeding in α-Syn-HEK-293 cells. ThT assay and TEM imaging demonstrated that lixisenatide slows α-Syn aggregation *in vitro*. α-Syn PFFs induced endogenous α-Syn aggregation and spreading between α-Syn-HEK-293 cells, but lixisenatide moderated the speed of α-Syn spread. In addition, we injected α-Syn PFFs into the striatum of wild-type mice to model sporadic PD. One week after ST injection, the mice began receiving lixisenatide treatment for 20 weeks. We found that lixisenatide alleviated motor dysfunction and reduced α-Syn phosphorylation and seeding, as well as neurodegeneration, in the α-Syn PFFs–induced PD mice model. Taken together, our findings provide new evidence for the neuroprotective effects of GLP-1R agonists and indicate that lixisenatide can hinder α-Syn aggregation and propagation.

To explore how lixisenatide inhibits α-Syn seeding, we tested the direct effects of lixisenatide on neurons. *In vivo* imaging showed that lixisenatide can cross BBB. α-Syn PFFs treatment increased GLP-1R and LAG3 expression in TH-positive neurons, while treatment with lixisenatide reversed this effect. Heparan sulfate proteoglycan, low-density lipoprotein receptor-related protein 1, and LAG3 are critical for the receptor-mediated transmission of pathological α-Syn (Pan and Peng, 2022; Luo et al., 2024). LAG3 has been demonstrated to bind to the C terminus of α-Syn fibrils, subsequently inducing their endocytosis (Angelopoulou et al., 2020; Zhang et al., 2021). Other studies have suggested that LAG3 can facilitate the transmission of α-Syn fibrils and worsen α-Syn pathology (Angelopoulou et al., 2020; Zhang et al., 2023). In this study, we discovered that lixisenatide may prevent α-Syn seeding via LAG3, which is expressed by neurons.

This study has some limitations that should be noted. First, the potential inhibitory effects of other GLP-1R agonists on α-Syn propagation were not examined. Future research should investigate other GLP-1R agonists have similar effects. Additionally, due to time constraints, neither LAG3 knockdown nor LAG3 overexpression were performed to fully establish the role of LAG3 in α-Syn propagation. Consequently, our assertion that LAG3 may play a critical role is speculative, based solely on the currently available evidence. In addition, mechanisms beyond LAG3 and mitochondrial protection should be considered in understanding how lixisenatide inhibits α-Syn propagation. Future research should prioritize exploring the mechanisms whereby GLP-1R agonists protect against PD.

In summary, lixisenatide protects against α-Syn pathology by inhibiting α-Syn aggregation and propagation in PD models, and this effect may be mediated by LAG3. This study offers new insights into the disease-modifying effects of GLP-1R agonists in the treatment of PD.

## Additional files:

***Additional Figure 1:*** *Lixisenatide alleviates α-Syn PFFs induced cell apoptosis and mitochondrial dysfunction in primary cultured neurons.*

Additional Figure 1 Lixisenatide alleviates α-Syn PFFs induced cell apoptosis and mitochondrial dysfunction in primary cultured neurons.
(A) PI (Red) and Hoechst 33342 (blue) double staining in the primary cultured neuron (scale bar: 50 μm). (B) Quantification of apoptosis rate in primary cultured neuron (n = 6 independent experiments). (C) Double staining of COX Ⅳ (red) and MAP2 (green) in the primary cultured neuron (scale bar: 100 μm). (D) Quantification of COX Ⅳ in primary cultured neuron (n = 6 independent experiments). Data were analyzed using Kruskal-Wallis test followed by Dunnett’s T3 multiple comparisons test (B) or one-way analysis of variance with Tukey’s multiple comparisons correction (D). The data are presented using a logarithmic transformation (D). Data are shown as mean ± SEM. *P < 0.05, **P < 0.01, ***P < 0.001. DAPI: 4′,6-Diamidino-2-phenylindole; Lix: lixisenatide; MAP2: microtubule associated protein 2; PI: propidium iodide; Veh: saline; α-Syn PFFs: α-synuclein preformed fibrils.

***Additional Figure 2:*** *Morphology of the peripheral organs of mice.*

Additional Figure 2 Morphology of the peripheral organs of mice.
(A) Transmission electron microscopic images of full-length α-Syn PFFs and short-ultrasonicated PFFs (scale bar: 200 nm). (B) HE staining of the peripheral organs including spleen, stomach, pancreas, liver, kidney and colon. The data showed that there is no morphology change in the peripheral organs after α-Syn PFFs and lixisenatide treatment (scale bar: 50 μm). Lix: Lixisenatide; Veh: saline; α-Syn PFFs: α-synuclein preformed fibrils.

## Data Availability

*All relevant data are within the paper and its Additional files.*
